# The Flow of Blood-Based Hybrid Nanofluids with Couple Stresses by the Convergent and Divergent Channel for the Applications of Drug Delivery

**DOI:** 10.3390/molecules26216330

**Published:** 2021-10-20

**Authors:** Anwar Saeed, Niqab Khan, Taza Gul, Wiyada Kumam, Wajdi Alghamdi, Poom Kumam

**Affiliations:** 1 Center of Excellence in Theoretical and Computational Science (TaCS-CoE), Faculty of Science, King Mongkut’s University of Technology Thonburi (KMUTT), 126 Pracha Uthit Rd., Bang Mod, Thung Khru, Bangkok 10140, Thailand; anwarsaeed769@gmail.com; 2 Mathematics Department, City University of Science and Information Technology, Peshawar 25000, Pakistan; niqabkhan85@gmail.com (N.K.); tazagul@cusit.edu.pk (T.G.); 3 Applied Mathematics for Science and Engineering Research Unit (AMSERU), Program in Applied Statistics, Department of Mathematics and Computer Science, Faculty of Science and Technology, Rajamangala University of Technology Thanyaburi, Thanyaburi, Pathumthani 12110, Thailand; 4 Department of Information Technology, Faculty of Computing and Information Technology, King Abdulaziz University, Jeddah 80261, Saudi Arabia; wmalghamdi@kau.edu.sa; 5 Department of Medical Research, China Medical University Hospital, China Medical University, Taichung 40402, Taiwan

**Keywords:** stretchable/shrinkable walls, hybrid nanofluid, drug delivery, couple stresses, Darcy–Forchheimer model, heat absorption/omission (HAM)

## Abstract

This research work aims to scrutinize the mathematical model for the hybrid nanofluid flow in a converging and diverging channel. Titanium dioxide and silver $\left(TiO_{2}\;\mathrm{and}\;Ag\right)$ are considered as solid nanoparticles while blood is considered a base solvent. The couple-stress fluid model is essentially use to describe the blood flow. Therefore, the couple-stress term was used in the recent study with the existence of a magnetic field and a Darcy–Forchheiner porous medium. The heat absorption/omission and radiation terms were also included in the energy equation for the sustainability of drug delivery. An endeavor was made to link the recent study with the applications of drug delivery. It has already been revealed by the available literature that the combination of $TiO_{2}$ with any other metal can destroy cancer cells more effectively than $TiO_{2}$ separately. Both the walls are stretchable/shrinkable, whereas flow is caused by a source or sink with α as a converging/diverging parameter. Governing equations were altered into the system of non-linear coupled equations by using the similarity variables. The homotopy analysis method (HAM) was applied to obtain the preferred solution. The influences of the modeled parameters have been calculated and displayed. The confrontation of wall shear stress and hybrid nanofluid flow increased as the couple stress parameter rose, which indicates an improvement in the stability of the base fluid (blood). The percentage (%) increase in the heat transfer rate with the variation of nanoparticle volume fraction was also calculated numerically and discussed theoretically.

## 1. Introduction

The flow of fluids in converging/diverging channels, such as flow in cavities and channels, has particularly important applications in science and engineering. The stretching, as well as the converging and diverging channels, corresponds to the blood flow in arteries and capillaries in the presence of the stress effect. Many studies have been conducted by using the flow of fluids in converging/diverging channels. Sheikholeslami et al. [1] demonstrated the effect of nanoparticles considering Jeffery fluid. It was noticed that the increasing values of Reynolds number caused a decline in inflow near the walls in the presence of a magnetic field. Convergent/divergent channels have useful applications: for example, in metal stream resistors, fiber manufacturing, production of plastic sheets, wire, and glasses. Turkyilmazoglu [2], Dogonchi and Ganji [3], Ahmed et al. [4], and Mishra et al. [5] have inspected the same model for the fluid flow using the concept of shrinking/stretching in converging/diverging channels.

Nanotechnology has been refined and expanded, the horizons of today’s scientific world owing to its surprising uses in various fields, for instance, therapeutics, medicines, biosciences, and drugs, etc. It has also been demonstrated that stenosis is damaging and causes fatal disease, so researchers attempted to eliminate the problem using nanotechnology. Researchers believe that nanotechnology can deliver innovation in treating these kinds of problems since nanoparticles can pass through tissues and cells. There is a noticeable rise in research related to the advanced progress of nanoparticles in drugs [6,7,8,9,10]. Choi [11] initiated the study of nanoparticles by revealing their dynamic and abnormal properties. Nadeem and Ijaz [12] described the use of nanoparticles to transport blood through a stenosis artery with a permeable wall. Ellahi et al. [13] reported blood flow to arteries consisting of the composite nanoparticles. Ijaz et al. [14] studied the effect of nanoparticles on stenotic artery hemodynamics and found them to be very helpful in reducing wall pressure with a shear rate.

The dispersion of more nanoparticles with different thermophysical properties from hybrid nanofluids attracted researchers because they are widely used in the fields of energy and medicine [15]. The field of bio nanotechnology has opened an innovative era in the field of medicine. This field has one of the most remarkable applications of hybrid nanofluids. Numerous studies have demonstrated the effectiveness of nanoparticles in tumor targeting, therapy, and diagnosis process. These studies have shown how effective nanoparticles are in tumor targeting, diagnosis, and treatment. It is worth mentioning that nanoparticles have eliminated some of the shortcomings of traditional chemotherapy [16]. Liu et al. [17] explored the use of $Pt/TiO_{2}$ and $Au/TiO_{2}$ nanocomposites, which are useful for cancer cell treatment. It was observed from the analysis that the combination of $TiO_{2}$ with any other metal can destroy more cancer cells than $TiO_{2}$ separately.

Silver has a wide range of biomedical uses due to its exclusive properties. Products containing silver are usually used for antimicrobial activity versus a broad spectrum of microorganisms. Moreover, experimental data suggest that Ag nanoparticles are a more ecological and biocompatible substitute to standard anticancer medicines [18].

Blood, the most important biological fluid, is a liquid composed of various cell types suspended in a matrix of aqueous fluid (the plasma). It should be noted that red blood cells in plasma contribute to rotary motion in the occurrence of a velocity gradient. Body tissues have an angular gyration moment as well as an angular orbital moment. As a result, blood may be assumed to be a non-Newtonian fluid with a constant density. Stokes’ theory is one of several polar fluid theories that can be taken into consideration [19].

Magnetohydrodynamic (MHD) applications in physiological problems are gaining popularity, and they are critical from both a theoretical and practical standpoint. Blood flow can be controlled with an adequate magnetic field. In 1936, the term “electromagnetic field” was first coined by Kollin [20] in medical research. Korchevskii and Marochnik [21] suggested the use of magnetic effects to regulate blood motion in the human system. Rao and Deshikachar [22] deliberate the influence of a transverse magnetic field on physiologic flux in an unvarying round tube. Vardanyan [23] demonstrated that the use of a magnetic field diminishes the velocity of blood motion. Therefore, all researchers reported that the use of magnetic influence decayed the blood flow [24]. The researchers used various methods to measure the blood flow and some of the techniques depend on the thermal properties to predict the temperature of the tissue. The temperature involvement in blood flow depends on the focal tissue temperature and thermal gradients. To sustain the uniform flow of blood and avoid the turbulence factor, researchers [25,26,27,28,29,30,31] used the temperature field in the blood flow analysis using various channels. The parameters involved in the energy equations vary in order to sustain the uniformity of blood flow in various circumstances and to improve the capability of drug delivery.

Health-related infections (IACs) are a major open well-being issue around the world, and TiO_2_ and Ag nanomaterials are used as antimicrobial for these kinds of infections. Moreover, the properties of TiO_2_ and Ag have valuable antimicrobial recognition and are utilized within Escherichia coli culture to assess their antibacterial viewpoint. The advancement within the temperature field gauges the pH values, and due to this reason, the TiO_2_ and Ag hybrid nanofluids are used for medicinal purposes.

In the light of the above discussion, the focus of this study is highlighted as follows:

- Until now, no one has investigated the flow through a converging/diverging stretchable/shrinkable channel with blood as a base fluid and $TiO_{2}\;\mathrm{and}\;Ag$ as nanoparticles.
- This article examines a suitable background of couple stress hybrid nanofluid flow through converging/diverging and stretchable/shrinkable channels.
- The addition of MHD, a Darcy–Forchheimer porous medium, thermal radiation, and heat absorption/omission terminologies further strengthen the novelty of the work.
- The system of equations was then analytically solved by HAM.

## 2. Formulation

**Figure 1:**
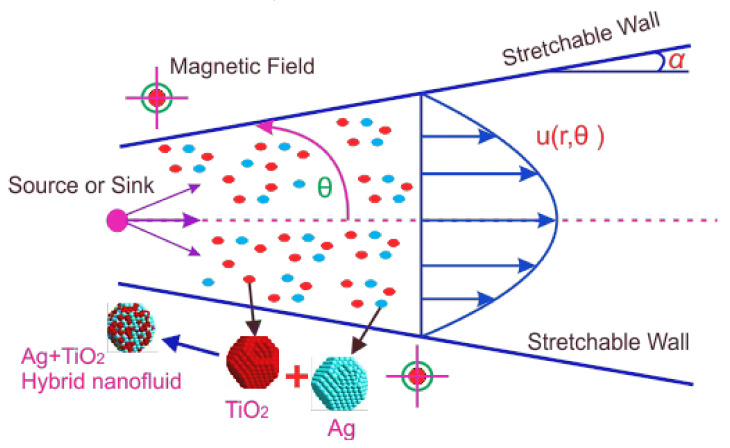
Geometry of the flow.

Assume the steady, laminar, and incompressible MHD hybrid nanofluid is set into motion by sink/source between two stretching/shrinking plates as shown in Figure 1. The angle between both is supposed to be 2α. It is further assumed that the walls of the channel are radially shrinkable as well as stretchable.

(1) $$u=\frac{s}{r}=u_{w},$$

where u is the velocity which is assumed to be redial, such that $u=u\left(r,\;\theta \right)$, whereas s depicts the stretching/shrinking rate. The surface temperature of the channel’s walls is *T*. The channel behaves in a divergent manner whenever α>0, otherwise the walls are converging. The magnetic effects are considered normal to the flow field, whereas the other assumptions of [25] are used; the basic constituent dimensional equations of the hybrid nanofluid are taken into account.

(2) $$\rho_{hnf}\left(\frac{u}{r}+\frac{\partial u}{\partial r}\right)=0,$$

(3) $$\begin{array}{r} \rho_{hnf}\left(u\frac{\partial u}{\partial r}\right)=-\left(\frac{\partial P}{\partial r}\right)+\mu_{hnf}\left(\frac{\partial^{2}u}{\partial r^{2}}+\frac{1}{r}\frac{\partial u}{\partial r}-\frac{u}{r^{2}}+\frac{1}{r^{2}}\frac{\partial^{2}u}{\partial \theta^{2}}\right)\\ -\eta_{0}\frac{\partial^{4}u}{\partial r^{4}}-\frac{C_{b}u^{2}}{\sqrt{k}}-\left(\frac{\mu_{hnf}}{K}+\frac{\sigma_{hnf}B_{0}{}^{2}}{r^{2}}\right)u, \end{array}$$

(4) $$\frac{\partial P}{\partial \theta }=2\mu_{hnf}\frac{1}{r^{2}}\frac{\partial u}{\partial \theta },$$

(5) $$\begin{array}{l} {\left(\rho C_{p}\right)}_{hnf}\left(u\frac{\partial T}{\partial r}\right)=k_{hnf}\left(\frac{\partial^{2}T}{\partial r^{2}}+\frac{1}{r^{2}}\frac{\partial^{2}T}{\partial \theta^{2}}+\frac{1}{r}\frac{\partial T}{\partial r}\right)+\mu_{hnf}\left(\frac{2u^{2}}{r^{2}}+2{\left(\frac{\partial u}{\partial r}\right)}^{2}+\frac{1}{r^{2}}{\left(\frac{\partial u}{\partial \theta }\right)}^{2}\right)+\\ \left(\frac{\sigma_{hnf}}{r^{2}}B_{0}{}^{2}u^{2}+\frac{Q_{0}}{r^{2}}T\right)-\left(\frac{1}{r^{2}}\frac{\partial }{\partial \theta }\left(q_{\theta ,rad}\right)+\frac{1}{r}\frac{\partial }{\partial r}\left(rq_{r,rad}\right)\right). \end{array}$$

The pressure of fluid, electromagnetic field, and radiative heat flux are presented by $P,B_{0},q_{r,rad},q_{\theta ,rad}$.

The radiation terms are further written as:

(6) $$q_{\theta ,rad}=\frac{-16\sigma^{*}T_{0}{}^{3}}{3k_{nf^{*}}}\frac{\partial T}{\partial \theta }\;\;\mathrm{and}\;\;q_{r,rad}=\frac{-16\sigma^{*}T_{0}{}^{3}}{3k_{nf^{*}}}\frac{\partial T}{\partial r}\;,$$

Here, $k_{nf^{*}},\sigma^{*}$ are the absorption terms and Stefan–Boltzmann constants.

Putting the values of Equation (6) into Equation (5), we have:

(7) $$\begin{array}{l} {\left(\rho C_{p}\right)}_{hnf}u\frac{\partial T}{\partial r}=\left(k_{hnf}+\frac{16\sigma^{*}T_{\infty }{}^{3}}{3k^{*}{}_{f}}\right)\left(\frac{\partial^{2}T}{\partial r^{2}}+\frac{1}{r^{2}}\frac{\partial^{2}T}{\partial \theta^{2}}+\frac{1}{r}\frac{\partial T}{\partial r}\right)\\ \;+\mu_{hnf}\left(2{\left(\frac{\partial u}{\partial r}\right)}^{2}\frac{1}{r^{2}}{\left(\frac{\partial u}{\partial \theta }\right)}^{2}+\frac{2u^{2}}{r^{2}}\right)+\left(\frac{\sigma_{hnf}}{r^{2}}B_{0}{}^{2}u^{2}+\frac{Q_{0}}{r^{2}}T\right), \end{array}$$

In the above expressions, the couple stress term is $\eta_{0}$, the Darcy–Forchheimer term is $C_{b}/\sqrt{k}$,$\mu_{hnf}$ is viscosity, $\rho_{hnf}$ is density, $\sigma_{hnf},\;k_{hnf}$ are electrical and thermal conductivities, ${\left(\rho C_{p}\right)}_{hnf}$ is specific heat. hnf describes hybrid nanofluid.

### 2.1. Thermophysical Properties

In the Table 1, Table 2 and Table 3, the thermophysical characteristics of nanofluid and hybrid nanofluid are described.

### 2.2. Initial and Boundary Conditions

The conditions at boundaries are:

(8) $$\left.\begin{array}{l} u=r^{-1}u_{c},\;\;\;\frac{\partial T}{\partial \theta }=u\frac{\partial T}{\partial r}=0,\;\;\;at\;\theta \to 0,\;r\neq 0\\ u=r^{-1}s=u_{w},\;\;T=r^{-2}T_{w}\;\;as\;\theta \to \pm \alpha \end{array}\right\}$$

### 2.3. Introduction of Non-Dimensional Variables

In case of radial flow, Equation (1) can be described as:

(9) f(θ)=r  u (r,  θ)

The non-dimensional transformation is defined as:

(10) $$f(\eta )={\left(u_{c}\right)}^{-1}f(\theta ),\;\;\Theta (\eta )=\left(r^{2}T\right)T_{w}^{-1},\;\;\eta =\theta \alpha^{-1},$$

In light of Equations (9) and (10) and Table 1, Table 2 and Table 3, Equations (3)–(5) are:

(11) f‴−μfμhnfρhnfρf2αReαReFr−1ff′−μfμhnfHa−4α2f′−24α2k∗f′−α2k1f′=0,

(12) Rd+khnfkfReΘ″+α2Re4+4Rd+ρCphnfρCpf2Prf+QΘ+ασhnfσfHaRePrEcf2+(1−ϕ1)−2.5(1−ϕ2)−2.5PrEcf′2+4α2f2=0,

The transformed boundary conditions in the shape of f(η) and θ(η) are:

(13) $$\begin{array}{l} \Theta^{\prime }(0)=f^{\prime }(0)=0,\;\;\;\;\;\;\;f(0)=1,\;\\ f(\pm 1)=\lambda ,\;\;\;\;\;\;\Theta (\pm 1)=1 \end{array}$$

In the above equations, $\lambda =\frac{s}{u_{c}}<0$ is the shrinking parameter, $\lambda =\frac{s}{u_{c}}>0$ is the stretching parameter, $Rd=\frac{16\sigma^{*}T^{3}{}_{\infty }}{3k_{f}k^{*}{}_{f}}$ is the radiation parameter, $K_{1}=\frac{\upsilon_{f}}{s\left(k\right)}$ is the porosity parameter, $\mathrm{Pr}=\frac{{\left(\mu C_{p}\right)}_{f}}{k_{f}}$ is the Prandtl number, $Ha=\frac{r\sigma_{f}B_{0}{}^{2}}{u_{c}\rho_{f}}$ is the Hartmann number, $Q=\frac{Q_{0}}{k_{f}}$ is the heat generation/absorption parameter, Re=rαucυf is the Reynolds number, $k^{*}=\frac{\eta_{0}}{\mu r^{2}}$ is the couple stress parameter, $Ec=\frac{u_{c}^{2}}{T_{w}{\left(C_{p}\right)}_{f}}$ is the Eckert number, and $Fr=\frac{C_{b}}{\sqrt{k}}r$ is the Darcy–Forchheimer parameter.

### 2.4. Rate of Heat Transfer Due to Drag Force

The rate of heat transmission along with drag force is described as:

(14) $$u_{c}^{2}\rho_{f}C_{f}={\left.\mu_{hnf}\left(\frac{1}{r}\frac{\partial u}{\partial \theta }\right)\right|}_{\theta =\pm \alpha },T_{w}Nu={\left.\left(\frac{16\sigma^{*}\;\;T^{3}{}_{\infty }}{3k_{f}\;\;k^{*}{}_{f}}+\frac{k_{hnf}}{k_{f}}\right)\frac{\partial T}{\partial \theta }\right|}_{\theta =\pm \alpha }.$$

In light of Equations (9) and (10), Equation (14) is

(15) ReCf=   μhnfμf  f′(±1)   ,    Nu=1αRd+khnfkfΘ′(±1).

## 3. Solution Methodology

Series solutions are one of the valuable methods to handle non-linear problems. The non-linear problems usually arise in the fields of engineering and sciences. HAM is one of the latest and fastest convergent techniques and is frequently used in the solution of non-linear and coupled equations. The BVPh. 1.0 and BVPh. 2.0 are the latest packages of HAM that enhance the convergence of the proposed problems. These packages are very helpful in the rapid convergence and the BVPh. 2.0 package up to the 100th iterations cannot be used easily. The idea of HAM was first introduced by Liao [32,33]. He has further improved the idea by introducing the packages [34]. These packages are frequently used for the solution of non-linear problems [35,36,37,38,39,40,41].

The proposed problem (11–16) was solved by the HAM-BVPh 2.0 technique. The estimate of the iterations was used up to the 30th order of approximations. The trial initial solutions are required for the HAM solution and are described as:

(16) $$f_{0}\left(\eta \right)=\left(\lambda -1\right)\eta^{2}+1,\;\theta_{0}(\eta )=1.$$

The Equations (11)–(13) are set by the proposed package and presented as:

(17) $${ƛ}_{l}^{f}=\frac{1}{m+1}\sum_{k=1}^{m}{\left[\prod_{F}{\left(\sum_{j=1}^{l}f\left(\eta \right)\right)}_{\zeta =kj\zeta }\right]}^{2},$$

(18) $${ƛ}_{l}^{\theta }=\frac{1}{m+1}\sum_{k=1}^{m}\;\;{\left[\prod_{\theta }\;\;{\left(\sum_{j=1}^{l}\theta \;\;\left(\eta \right)\right)}_{\zeta =kj\zeta },{\left(\sum_{j=1}^{l}\;\;f\left(\eta \right)\right)}_{\zeta =kj\zeta }\;\;\right]}^{2},$$

The sum of the two components in the form of square residual errors is displayed as:

(19) $${ƛ}_{l}^{Total}={ƛ}_{l}^{f}+{ƛ}_{l}^{\theta }.$$

The numerical results of the converging parameter are $0.130021\leq h_{f}\leq -1.203417,0.120432\leq h_{\theta }\leq -0.8992310.$

The range of convergence control parameters was used to find out the physical and numerical results.

## 4. Discussion of Results

**Figure 2:**
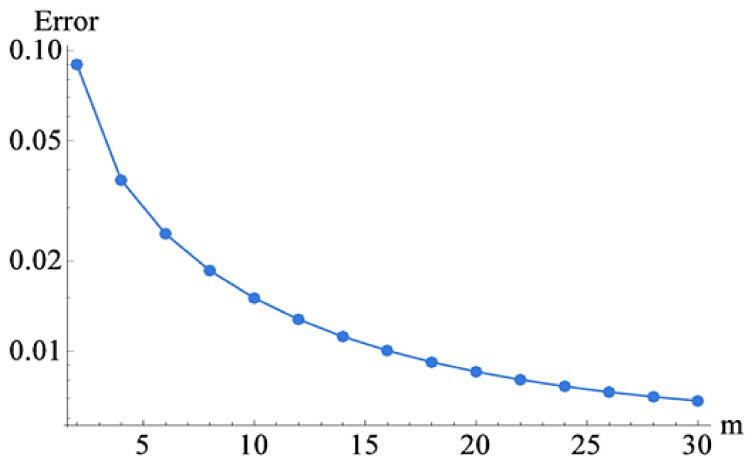
The convergence approach up to 30th order iterations.

The flow of the blood-based hybrid nanofluid consisting of $TiO_{2}\;\mathrm{and}\;Ag$ was considered in the converging and diverging channels. The heat transfer mechanism and medication are the main purposes of the proposed model. The main findings of the obtained results are shown physically and numerically. Figure 1 shows the geometry of the problem. Figure 2 shows the convergence of the HAM technique.

The titanium dioxide and silver nanoparticles were dispersed in the bloodstream (base fluid) to synthesize the hybrid nanofluid. $TiO_{2}$ represents Titanium dioxide nanomaterial, Ag describes Silver nanoparticles and subscript f describes blood (base fluid). In Table 1 and Table 2, $\phi_{1}$ and $\phi_{2}$ illustrate the volume fractions of $TiO_{2}$ and Ag nanoparticles in the base fluid, where $\phi_{1}=\phi_{2}=0$ refers to the simple base fluid. Similarly, the experimental results of the materials are presented in Table 3. Table 4 shows the comparison of the existing results with published literature, where the closed agreement authenticates the validation of the problem. The values of skin friction for the variations in parameters were determined numerically and are depicted in Table 5. By applying the concept of convergence/divergence approaches, the values of skin friction were determined over the surface of the walls. It was observed that the value of skin friction rose with the enhancement of different parameters at both the lower and upper walls. In the case of hybrid nanofluid, the skin friction also improved more than in the traditional nanofluid. It was observed that the skin friction had higher values in the case of the converging channel than in the case of the diverging channel. By incorporating the concept of diverging and converging channels, the numerical values of the Nusselt number were determined at different values of parameters for hybrid and traditional nanofluids and are presented in Table 6. It was reported that with rising values of Rd,Ec,Q, there is an augmentation in the thermal transmission rate. This augmentation is comparatively greater in the case of hybrid nanofluid than traditional nanofluid. It was also perceived that the Nusselt number increased more efficiently in the case of a convergent channel than in a divergent one. Moreover, silver is often used for stabilizing the blood, whereas $TiO_{2}$ is applied for the treatment of cancer. Table 7 depicts the percentage augmentation in the thermal flow rate against nanoparticles volumetric fraction, which was observed to be augmented more for hybrid nanofluid than traditional nanofluid.

Figure 3, Figure 4, Figure 5, Figure 6, Figure 7, Figure 8, Figure 9, Figure 10, Figure 11, Figure 12, Figure 13 and Figure 14 portray the influence of $\phi_{1},\;\;\phi_{2},\;Ha,\;\;k_{1},\;\;Fr,\;Re\;\;\mathrm{and}\;\;k^{*}$ on velocity profiles for converging/stretching and diverging/shrinking channels.

**Figure 3:**
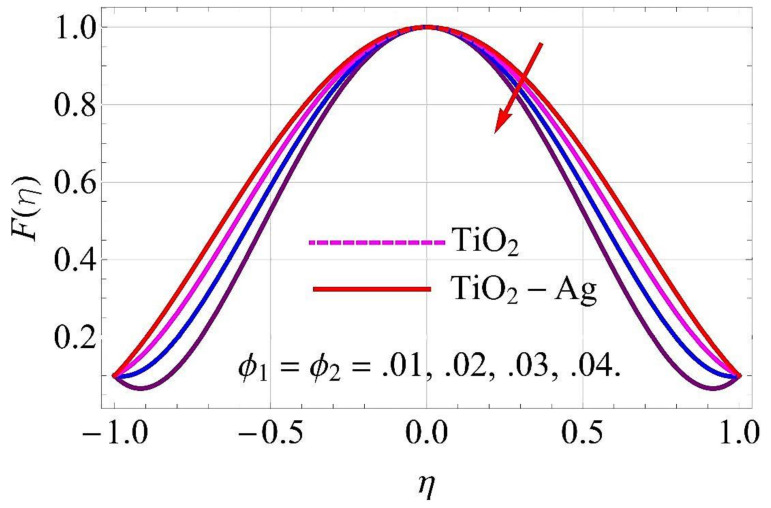
Velocity versus $\phi_{1},\phi_{2}$ with $\alpha =-5^{\circ },$ for converging/stretching circumstances. When Re=25,Rd=0.4,Ec=Ha=Q=Fr=k*=K1=0.1.

**Figure 4:**
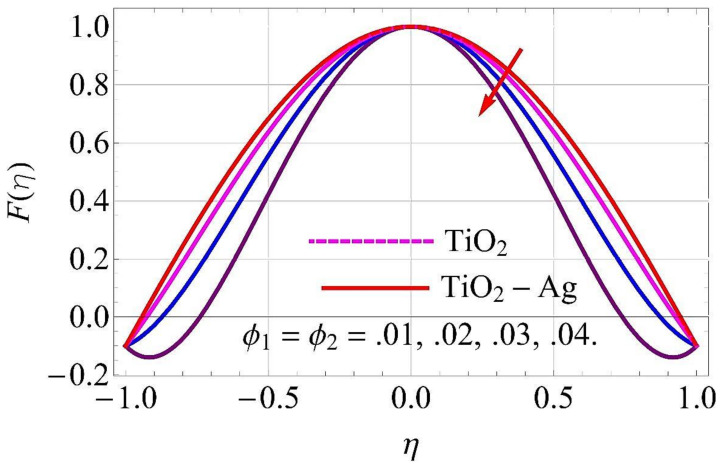
Velocity versus $\phi_{1},\phi_{2}$ with $\alpha =5^{\circ }$ for diverging/shrinking circumstances. When Re=25,Rd=0.4,Ec=Ha=Q=Fr=k*=K1=0.1.

The influence of the volumetric fractions of $TiO_{2}$ and Ag nanoparticles on flow for converging/stretching and diverging/shrinking walls of the channel are depicted in Figure 3 and Figure 4, respectively. In both cases, it was observed that the fluid flow reduced with the rising values of the volumetric fraction. When the nanoparticles were exposed to pure fluid, its density enhanced, due to which fluid became denser and caused a decay in the flow through the channel.

**Figure 5:**
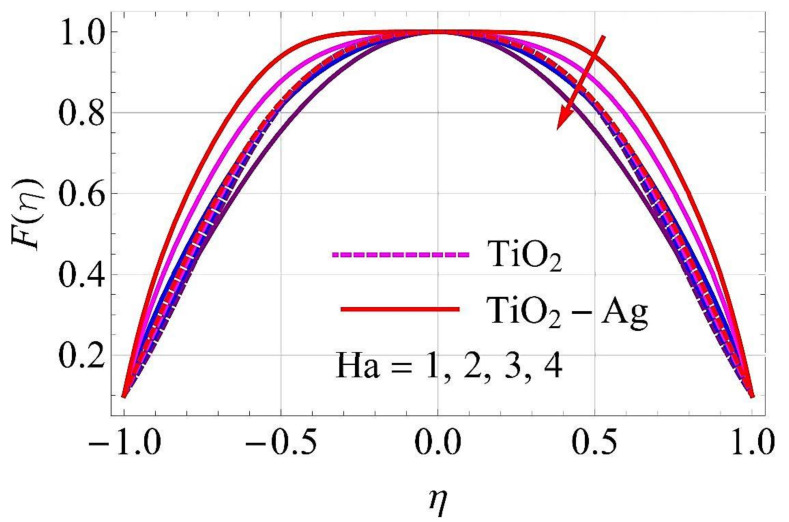
Velocity versus Ha with $\alpha =-5^{\circ }$ for converging/stretching circumstances. When Re=25,ϕ1=ϕ2=0.01,Rd=0.4,Ec=Q=Fr=k*=K1=0.1.

**Figure 6:**
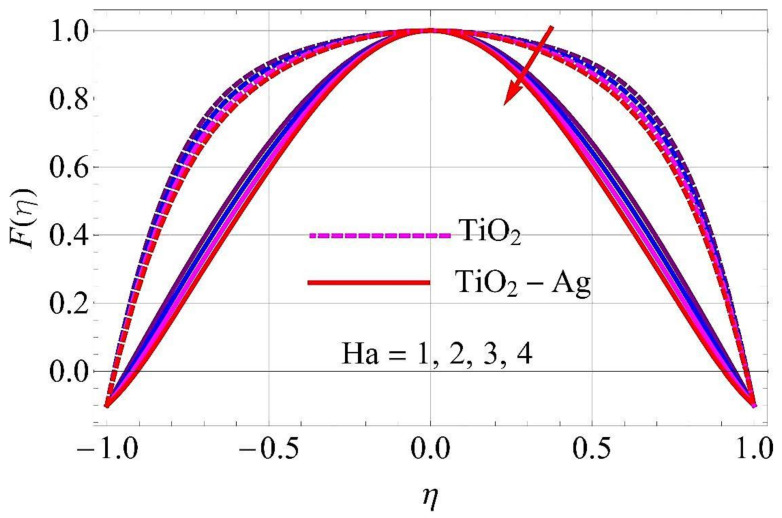
Velocity versus Ha with $\alpha =5^{\circ }$ for diverging/shrinking circumstances. When Re=25,ϕ1=ϕ2=0.01,Rd=0.4,Ec=Q=Fr=k*=K1=0.1.

Figure 5 and Figure 6 deliberate the effects on flow for converging/stretching and diverging/shrinking walls of the channel in response to variation in the Hartmann number *Ha*. With the rising trend of *Ha*, the Lorentz force was generated, which acted against the flow direction. As a result, the fluid velocity declined in both cases.

**Figure 7:**
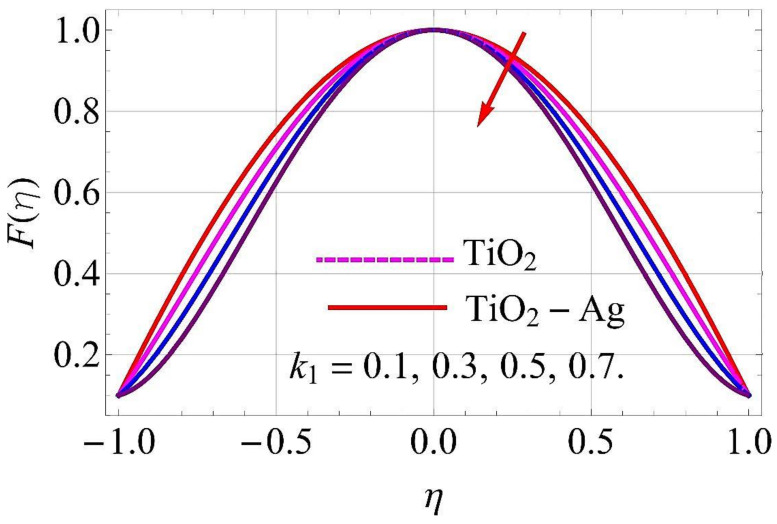
Velocity versus $k_{1}$ with $\alpha =-5^{\circ }$ for converging/stretching circumstances. When Re=25,ϕ1=ϕ2=0.01,Rd=0.4,Ec=Q=Fr=k*=Ha=0.1.

**Figure 8:**
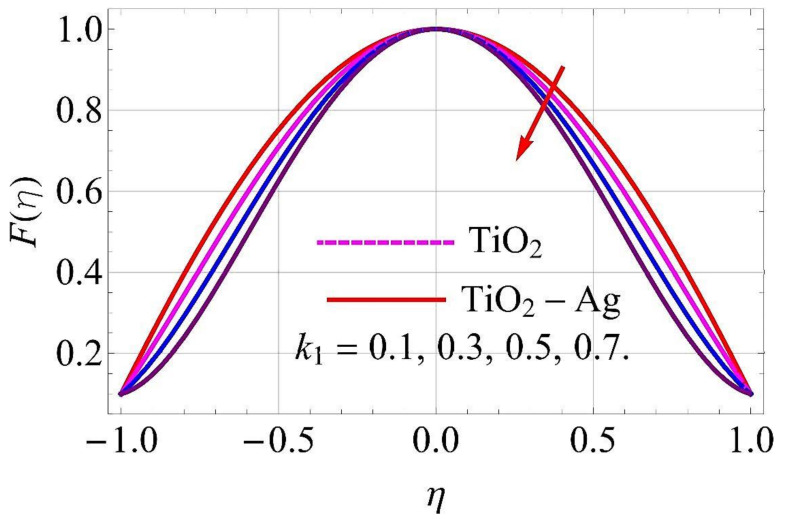
Velocity versus $k_{1}$ with $\alpha =5^{\circ }$ for diverging/shrinking circumstances. When Re=25,ϕ1=ϕ2=0.01,Rd=0.4,Ec=Q=Fr=k*=Ha=0.1.

Figure 7 and Figure 8 portray that the augmenting values of the porosity parameter $k_{1}$ correspond to a decline in the flow of fluid. Physically, it can be interpreted that with the increment in $k_{1}$, the void spaces in the medium enhanced, and more resistance was offered to the flow profile in both cases of the channel. For this reason, the fluid motion was reduced.

**Figure 9:**
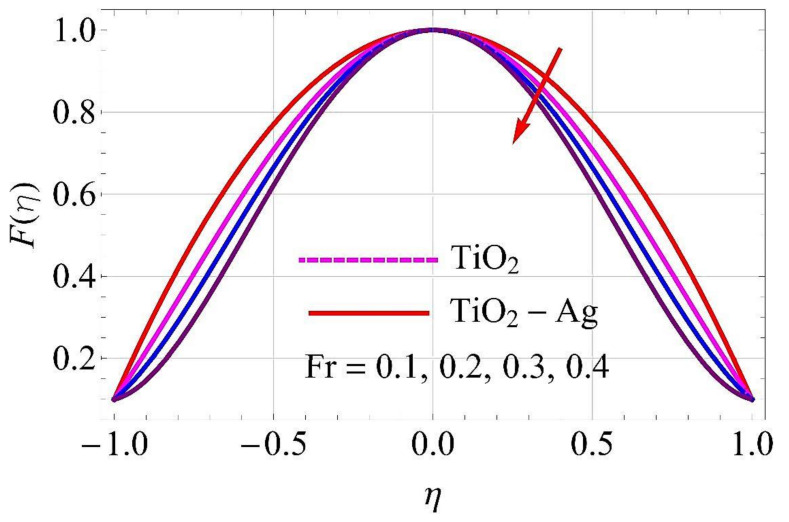
Velocity versus Fr with $\alpha =-5^{\circ }$ for converging/stretching circumstances. When Re=25,ϕ1=ϕ2=0.01,Rd=0.4,Ec=Q=Ha=k*=K1=0.1.

**Figure 10:**
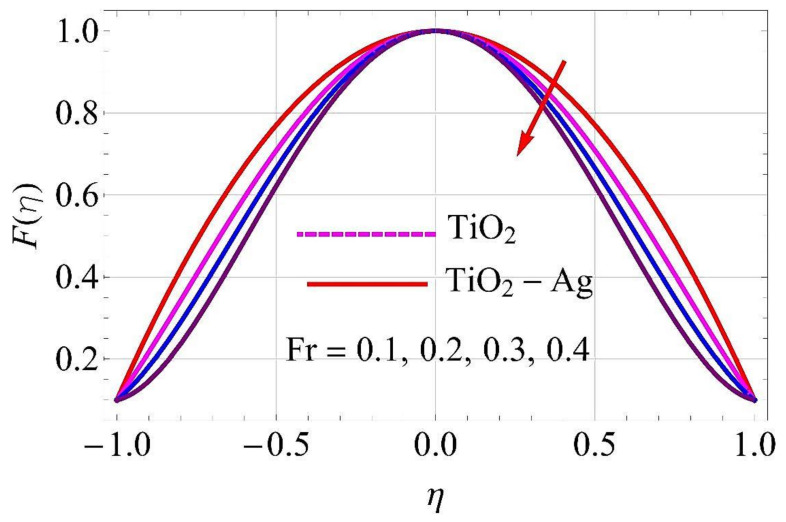
Velocity versus Fr with $\alpha =5^{\circ }$ for diverging/shrinking circumstances. When Re=25,ϕ1=ϕ2=0.01,Rd=0.4,Ec=Q=Ha=k*=K1=0.1.

Figure 9 and Figure 10 depict the influence of Forchheimer parameter *Fr* on flow for converging/stretching and diverging/shrinking walls of the channel. It was noticed from these figures that for augmentation in *Fr* there was more resistance experienced by the fluid particles. In this physical phenomenon, the flow declined both for converging/stretching and diverging/shrinking walls.

**Figure 11:**
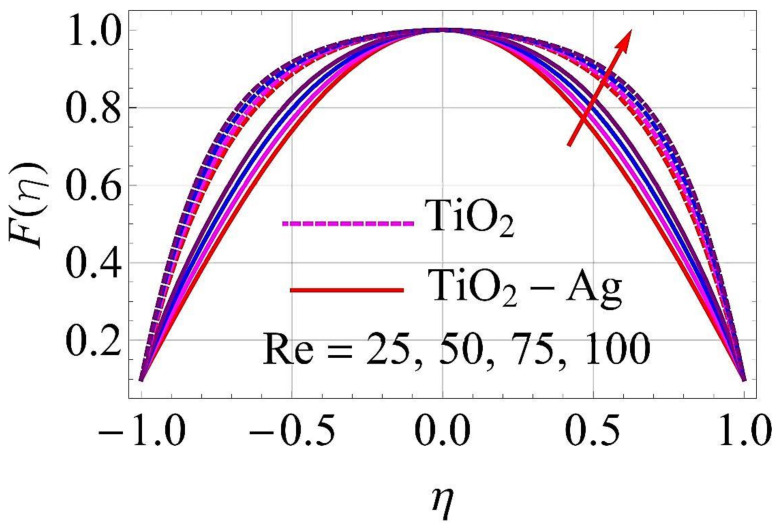
Velocity versus Re with $\alpha =-5^{\circ }$ for converging/stretching circumstances. When $\phi_{1}=\phi_{2}=0.01,Rd=0.4,Ec=Q=Ha=k^{*}=Fr=K_{1}=0.1$.

**Figure 12:**
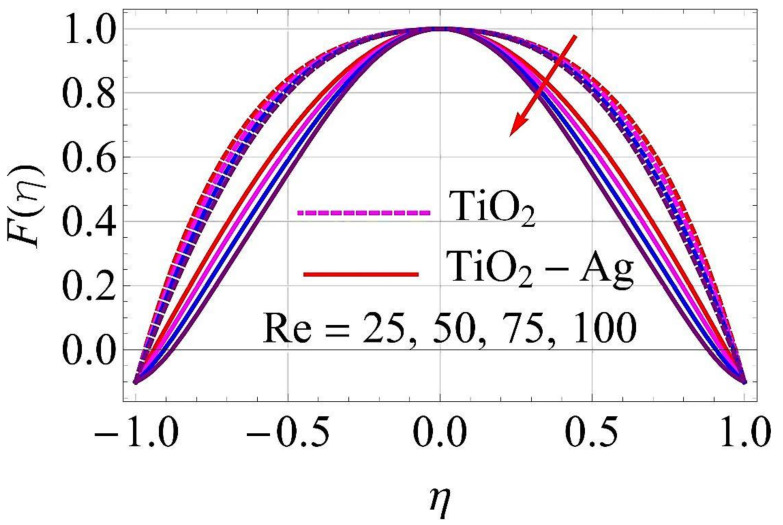
Velocity versus Re with $\alpha =5^{\circ }$ for diverging/shrinking circumstances. When $\phi_{1}=\phi_{2}=0.01,Rd=0.4,Ec=Q=Ha=k^{*}=Fr=K_{1}=0.1$.

It was observed from Figure 11 and Figure 12 that the augmentation in Reynolds number Re had a different response for the flow of fluid. For higher values of Re, the flow reached its maximum in case of converging and stretching in the surface of the walls, as depicted in Figure 11. In the case of diverging and shrinking walls, the impact on flow is in reverse for growing values of Re, as shown in Figure 12.

**Figure 13:**
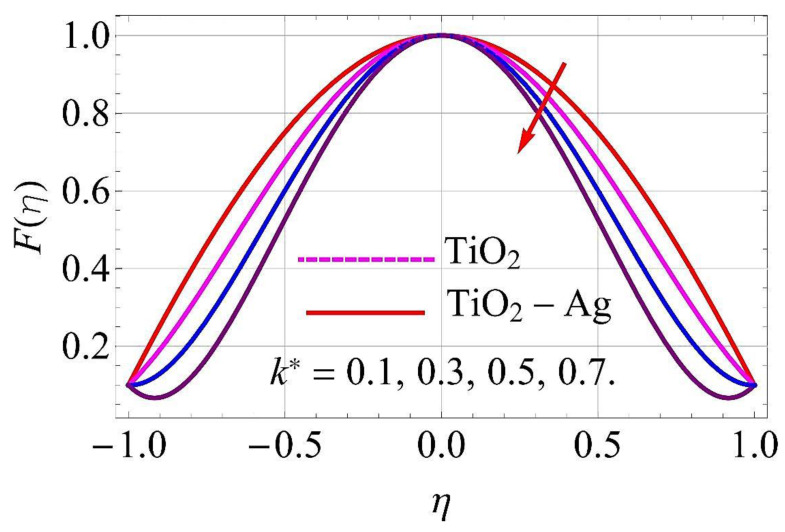
Velocity versus $k^{*}$ with $\alpha =-5^{\circ }$ for converging/stretching circumstances. When Re=25,ϕ1=ϕ2=0.01,Rd=0.4,Ec=Q=Ha=k*=Fr=0.1.

**Figure 14:**
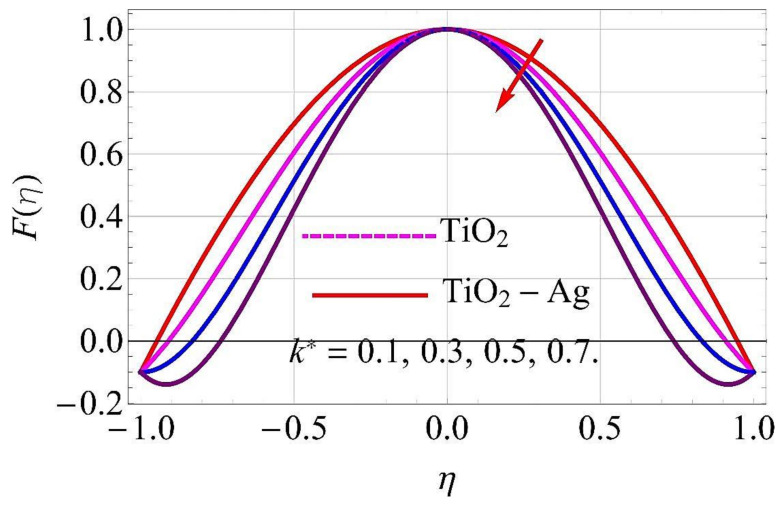
Velocity versus $k^{*}$ with $\alpha =5^{\circ }$ for diverging/shrinking circumstances. When $\phi_{1}=\phi_{2}=0.01,Rd=0.4,Ec=Q=Ha=k^{*}=Fr=K_{1}=0.1$.

With the augmentation in couple stress parameter $k^{*}$, the fluid boundary layer strength weakened in both diverging/shrinking as well as in converging/stretching walls of the channel. Due to this, the flow of fluid declined in both cases, as depicted in Figure 13 and Figure 14.

Figure 15, Figure 16, Figure 17, Figure 18, Figure 19, Figure 20, Figure 21, Figure 22, Figure 23 and Figure 24 present the influences on the thermal profiles in response to different parameters, such as $\alpha ,\;\;Ha,\;\;Ec,\;\;Q,\;\;Pr,\;\;\mathrm{and}\;\;(\phi_{1},\phi_{2})$ for converging/stretching and diverging/shrinking walls of the channel.

**Figure 15:**
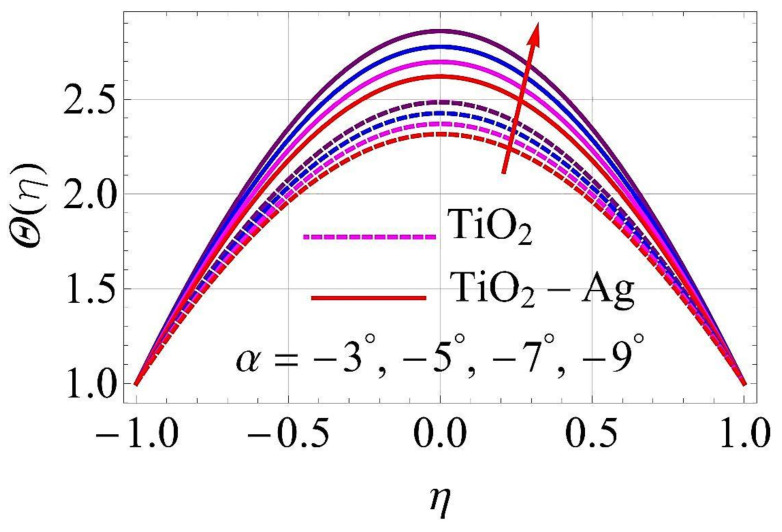
Temperature versus variations in α for converging/stretching circumstances. When Re=25,ϕ1=ϕ2=0.01,Rd=0.4,Ec=Q=Ha=k*=Fr=K1=0.1.

**Figure 16:**
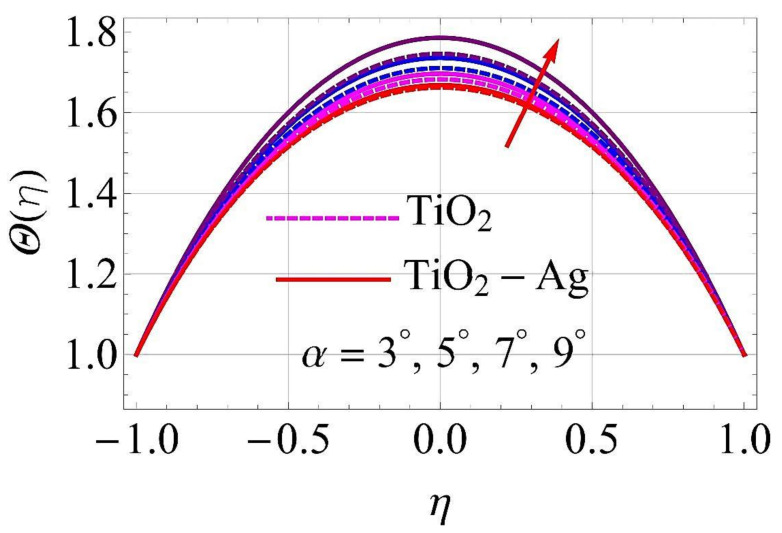
Temperature versus variations in α for diverging/shrinking circumstances. When Re=25,ϕ1=ϕ2=0.01,Rd=0.4,Ec=Q=Ha=k*=Fr=K1=0.1.

Figure 15 and Figure 16 portray the influence on thermal profiles for the augmentation of the values of the diverging and converging parameter α. It was observed from these figures that the thermal layer must strengthen with higher values of α. Hence, the thermal profiles were augmented with enhancing values of α in both cases at walls of the channel.

**Figure 17:**
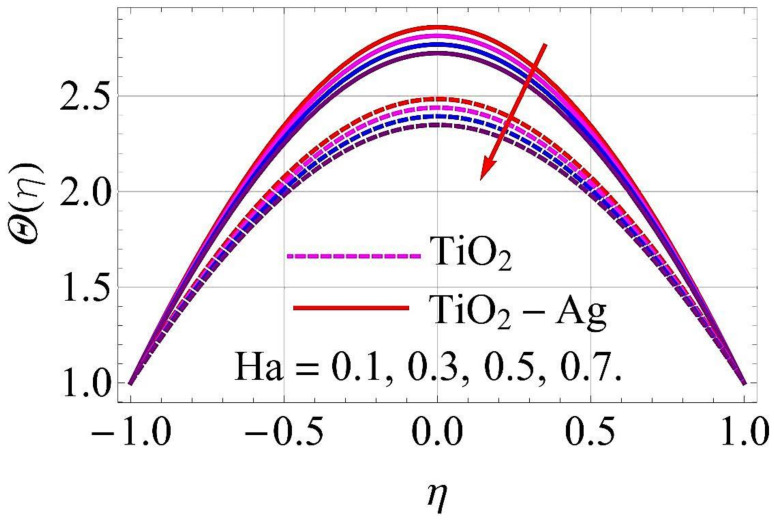
Temperature versus Ha with $\alpha =-5^{\circ }$ for converging/stretching circumstances. When Re=25,ϕ1=ϕ2=0.01,Rd=0.4,Ec=Q=k*=Fr=K1=0.1.

**Figure 18:**
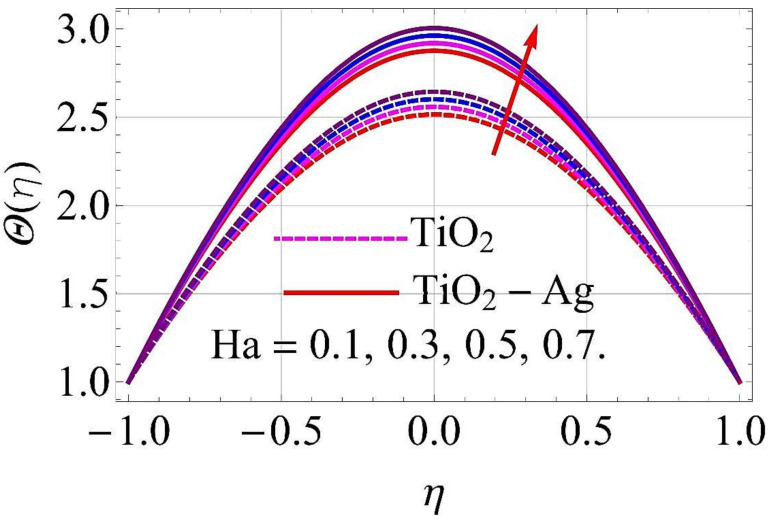
Temperature versus Ha with $\alpha =5^{\circ }$ for diverging/shrinking circumstances. When Re=25,ϕ1=ϕ2=0.01,Rd=0.4,Ec=Q=k*=Fr=K1=0.1.

The influence of the Hartmann number Ha on thermal profile in the case of converging/stretching and diverging/shrinking surfaces of the walls is depicted in Figure 17 and Figure 18. For maximum growth in Ha, the thermal profiles decayed when the walls were converging/stretching, as revealed in Figure 17. The thermal profile was observed to be augmented with growth in Ha for diverging/shrinking cases, as depicted in Figure 18.

**Figure 19:**
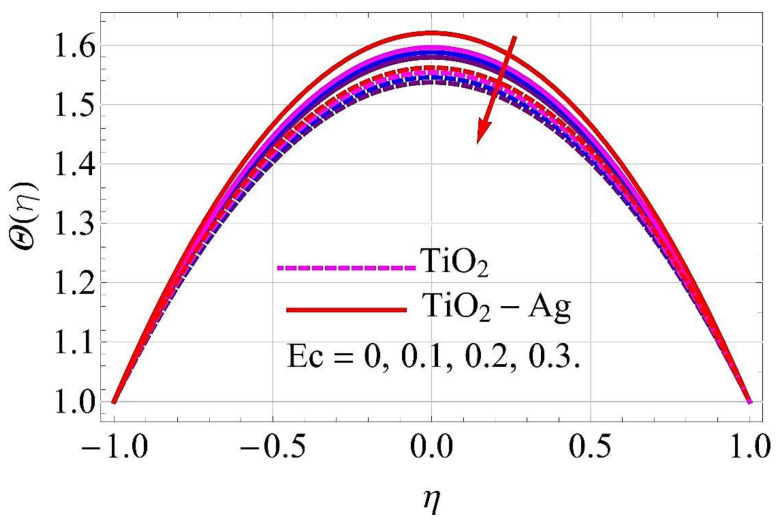
Temperature versus Ec with $\alpha =-5^{\circ }$ for converging/stretching circumstances. When Re=25,ϕ1=ϕ2=0.01,Rd=0.4,Q=Ha=k*=Fr=K1=0.1.

**Figure 20:**
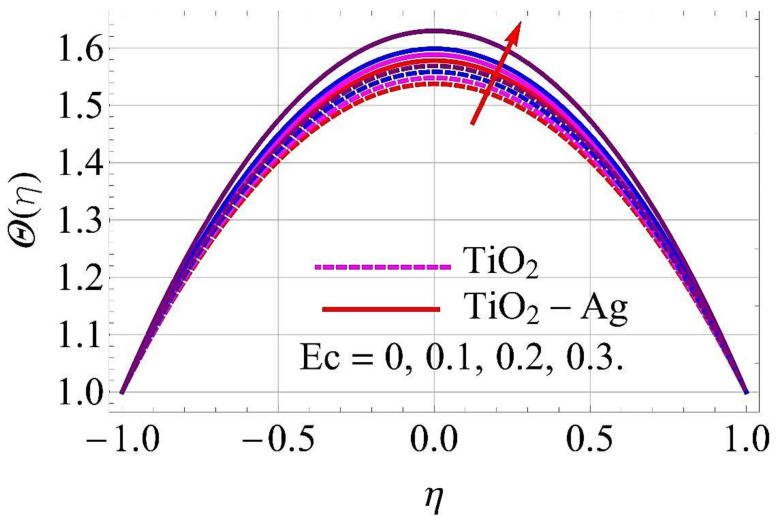
Temperature versus Ec with $\alpha =5^{\circ }$ for diverging/shrinking circumstances. When Re=25,ϕ1=ϕ2=0.01,Rd=0.4,Q=Ha=k*=Fr=K1=0.1.

Figure 19 and Figure 20 portray the impact of the Eckert number Ec on thermal profile. From Figure 20, it can be observed that with an augmentation in Ec, the fluid is resisted more than enhanced by the temperature of the fluid flow. However, there was an adverse impact in the case of converging and stretching the surface of the channel, as depicted in Figure 19.

**Figure 21:**
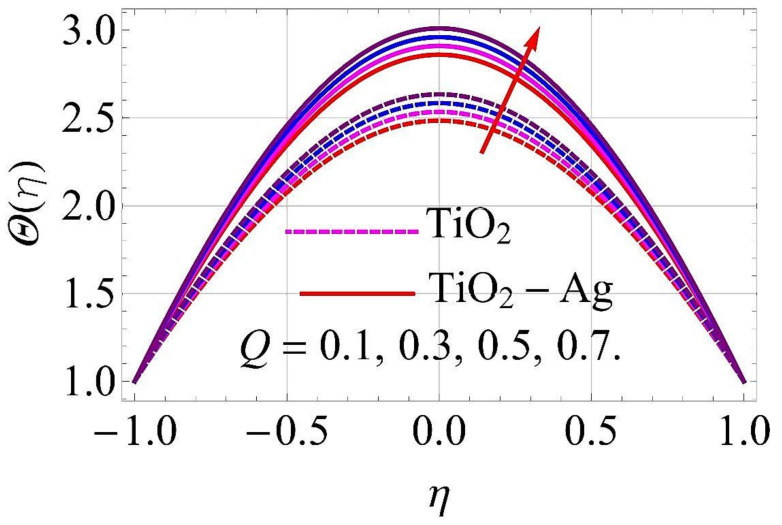
Temperature versus Q with $\alpha =-5^{\circ }$ for converging/stretching circumstances. When Re=25,ϕ1=ϕ2=0.01,Rd=0.4,Ec=Ha=k*=Fr=K1=0.1.

**Figure 22:**
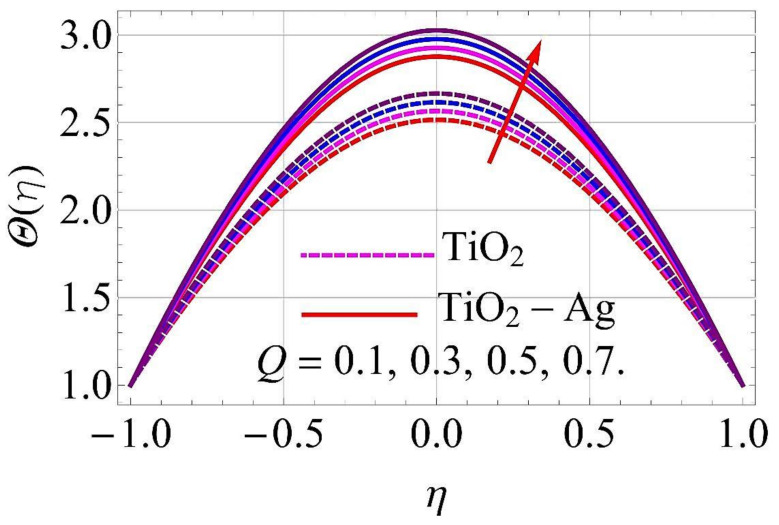
Temperature versus Q with $\alpha =5^{\circ }$ for diverging/shrinking circumstances. When Re=25,ϕ1=ϕ2=0.01,Rd=0.4,Ec=Ha=k*=Fr=K1=0.1.

Figure 21 and Figure 22 present the variations in thermal profiles in response to variations in omission/absorption parameter Q for converging/stretching and diverging/shrinking walls. It was observed that for growth in the values of Q, additional heat was included in the flow system, which enhanced the thermal profiles for both cases.

**Figure 23:**
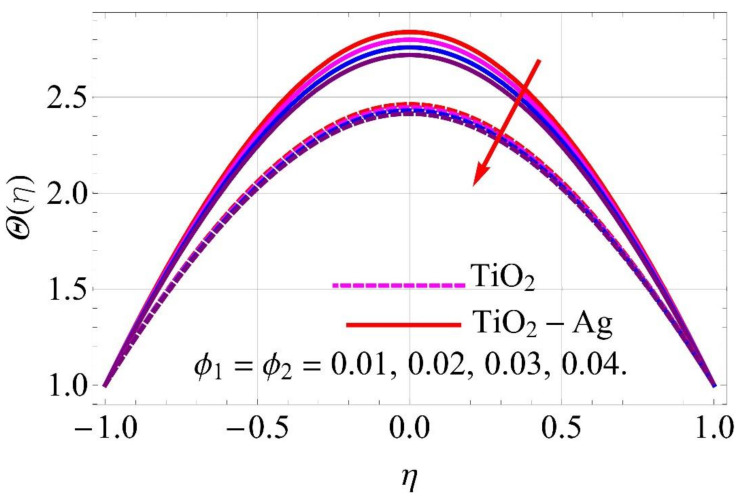
Temperature versus $\phi_{1},\phi_{2}$ with $\alpha =-5^{\circ }$ converging/stretching circumstances.

**Figure 24:**
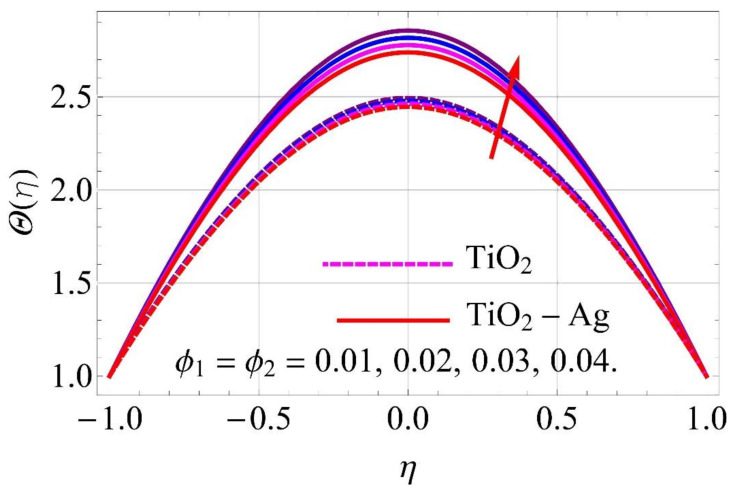
Temperature versus $\phi_{1},\phi_{2}$ with $\alpha =5^{\circ }$ diverging/shrinking circumstances. When Re=25,ϕ1=ϕ2=0.01,Rd=0.4,Ec=Q=Ha=k*=Fr=K1=0.1.

Figure 23 and Figure 24 depict the impact of volumetric fractions of nanoparticles on thermal profiles. It can be observed from Figure 24 that with the addition of nanoparticles to pure fluid, the denser behavior of fluid was enhanced, causing an augmentation in the thermal profile for diverging/shrinking walls. An adverse impact was noticed in the case of converging/stretching case, as depicted in Figure 23.

## 5. Conclusions

The current article investigated the blood flow through a converging/diverging channel with stretchable/shrinkable walls with couple stress for the application of drug delivery through a porous medium under the effect of MHD. The effects of the converging/diverging parameter, Hartmann number, heat generation/absorption parameter, Eckert number, Prandtl number, porosity parameter, couple stress parameter, Darcy–Forchheimer parameter, and solid volume fraction were incorporated. The properties of TiO2 and Ag have valuable antimicrobial recognition and were utilized within Escherichia coli culture to assess their antibacterial viewpoint. The advancement within the temperature field gauges the pH values and due to this reason, the TiO2 and Ag hybrid nanofluids are used for medicinal purposes.

The key conclusions of the existing study are as follows:

- The rising value of $Ec,Q,\phi_{1},\phi_{2}$ increases the temperature field and this impact is relatively larger in the case of hybrid nanofluid.
- The velocity field declines with the accumulative values of the parameters ϕ1,ϕ2,Fr, k1,Re.
- The Hartmann number has a significant role in blood flow analysis. The strong magnetic field declines the hybrid nanofluid motion.
- $TiO_{2}\;\mathrm{and}\;Ag$ hybrid nanofluids have an important role in Escherichia coli culture to evaluate their antibacterial strength.
- The electric conductivity and pH values improve with the increment in heat transfer. Therefore, the purpose of the recent study was to use the $TiO_{2}+Ag$ hybrid nanofluids for medication.

## 6. Future Suggestions

- The researcher can use the slip conditions for the same model.
- The current study could be extended to consider other nanoparticles and fluids for industrial uses.
- The same model could be extended to include concentration and bioconvection microorganisms.
- Other numerical and analytical methods can extend the present work by using the comparative analysis.

## Figures and Tables

**Table 1:** Thermophysical properties of $TiO_{2}-Blood$ nanofluid [3,18].

Viscosity	$\mu_{nf}=\frac{\mu_{f}}{{\left(1-\phi_{1}\right)}^{2.5}}$
Density	$\rho_{nf}=\left\{\rho_{f}(1-\phi_{1})+\rho_{f}\phi_{1}\left(\rho_{TiO_{2}}\right)\right\}$
Specific Heat	${\left(\rho C_{p}\right)}_{nf}=\left[{\left(\rho C_{p}\right)}_{f}(1-\phi_{1})+\phi_{1}\left({\left(\rho C_{p}\right)}_{TiO_{2}}\right)\right]$
Thermal Conductivity	$k_{nf}\;=k_{f}{\left(k_{TiO_{2}}+2k_{f}+2\phi_{1}(k_{f}-k_{TiO_{2}})\right)}^{-1}\left(k_{TiO_{2}}+2k_{f}-2\phi_{1}(k_{f}-k_{TiO_{2}})\right)$

**Table 2:** Various thermophysical properties of $TiO_{2}-Ag/Blood$ are stated as [18,26].

Viscosity	$\mu_{hnf}=\mu_{f}{\left(1-\phi_{1}\right)}^{-2.5}{\left(1-\phi_{2}\right)}^{-2.5}$
Density	$\rho_{hnf}=(1-\phi_{2})\left\{\phi_{1}\rho_{TiO_{2}}+(1-\phi_{1})\;\rho_{f}\right\}+\phi_{2}\rho_{TiO_{2}}$
Specific Heat	${\left(\rho C_{p}\right)}_{hnf}={\left(\rho C_{p}\right)}_{f}(1-\phi_{2})(1-\phi_{1})+\phi_{1}\left({\left(\rho C_{p}\right)}_{TiO_{2}}\right)+\phi_{2}\left({\left(\rho C_{p}\right)}_{Ag}\right)$
Thermal Conductivity	$\begin{array}{l} k_{hnf}=k_{f}\left\{{\left(k_{TiO_{2}}+2k_{nf}+2\phi_{2}(k_{nf}-k_{TiO_{2}})\right)}^{-1}\left(k_{TiO_{2}}+2k_{nf}-2\phi_{2}(k_{nf}-k_{TiO_{2}})\right)\right\}\times \\ \left\{{\left(k_{Ag}+2k_{f}-2\phi_{1}(k_{f}-k_{Ag})\right)}^{-1}\left(k_{Ag}+2k_{f}-2\phi_{1}(k_{f}-k_{Ag})\right)\right\}, \end{array}$

**Table 3:** Numerous thermophysical properties are defined as [18].

**Solid Material and Base Fluid**	$c_{p}(J/\mathrm{kgK})$	k(W/mK)	$\rho (Kg/m^{3})$
$TiO_{2}$ **(Titanium Dioxide)**	686.2	8.954	4250
**Silver:** Ag	235	429	10,500
**Blood**	3594	0.492	1063

**Table 4:** Comparison between the present work and previous work considering common parameters only.

Re	$\begin{array}{l} f^{\prime \prime }(\pm 1),\\ \left(\alpha =5^{{}^{\circ}}\right)\\ \left[3\right] \end{array}$	$\begin{array}{l} f^{\prime \prime }(\pm 1)\\ \left(\alpha =5^{{}^{\circ}}\right)\\ \left[4\right] \end{array}$	$\begin{array}{l} f^{\prime \prime }(\pm 1)\\ \left(\alpha =5^{{}^{\circ}}\right)\\ \left[5\right] \end{array}$	$\begin{array}{l} f^{\prime \prime }(\pm 1)\\ \left(\alpha =5^{{}^{\circ}}\right)\\ \left[\mathrm{Present}\right] \end{array}$	$\begin{array}{l} f^{\prime \prime }(\pm 1),\\ \left(\alpha =-5^{{}^{\circ}}\right)\\ \left[3\right] \end{array}$	$\begin{array}{l} f^{\prime \prime }(\pm 1)\\ \left(\alpha =-5^{{}^{\circ}}\right)\\ \left[4\right] \end{array}$	$\begin{array}{l} f^{\prime \prime }(\pm 1)\\ \left(\alpha =-5^{{}^{\circ}}\right)\\ \left[5\right] \end{array}$	$\begin{array}{l} f^{\prime \prime }(\pm 1)\\ \left(\alpha =-5^{{}^{\circ}}\right)\\ \left[\mathrm{Present}\right] \end{array}$
1	1.8642	1.8643	1.8641	1.86401	0.7742	0.7743	0.7741	0.7740
2	1.8864	1.8865	1.8863	1.8861	0.7953	0.7954	0.7952	0.7952
3	1.90422	1.9043	1.9041	1.9040	0.8021	0.8022	0.8020	0.8020

**Table 5:** The skin friction $-ReC_{f}\;$ versus different parameters.

$\phi_{1},\phi_{2}$	$K_{1}$	Ha	Re	$k^{*}$	Fr	$\begin{array}{l} -ReC_{f}\;\\ \left(\alpha >0\right)\\ TiO_{2}+Ag \end{array}$	$\begin{array}{l} -ReC_{f}\;\\ \left(\alpha >0\right)\\ TiO_{2} \end{array}$	$\begin{array}{l} -ReC_{f}\;\\ \left(\alpha >0\right)\\ TiO_{2}+Ag \end{array}$	$\begin{array}{l} -ReC_{f}\;\\ \left(\alpha >0\right)\\ TiO_{2} \end{array}$
0.01	0.1	0.1	0.1	0.1	0.1	0.392	0.371	1.292	1.273
0.02						0.416	0.404	1.268	1.243
0.03						0.432	0.411	1.284	1.263
	0.3					0.532	0.512	1.421	1.402
	0.5					0.746	0.724	1.631	1.614
		0.3				0.435	0.413	1.324	1.303
		0.5				0.513	0.501	1.401	1.393
			0.3			0.494	0.472	1.383	1.361
			0.5			0.595	0.573	1.493	1.271
				0.3		0.464	0.442	1.353	1.127
				0.5		0.512	0.501	1.402	1.383
					0.3	0.422	0.401	1.311	1.301
					0.5	0.633	0.611	1.522	1.502

**Table 6:** Nusselt number $Nu_{x}$ versus different parameters.

Rd	Ec	Q	$\begin{array}{l} -Nu(\alpha >0)\\ TiO_{2}+Ag \end{array}$	$\begin{array}{l} -Nu\;(\alpha >0)\\ TiO_{2} \end{array}$	$\begin{array}{l} -Nu\;(\alpha <0)\\ TiO_{2}+Ag \end{array}$	$\begin{array}{l} -Nu(\alpha <0)\\ TiO_{2} \end{array}$
0.1	0.1	0.1	9.416	9.331	11.393	11.292
0.3			9.474	9.373	11.503	11.322
0.5			9.537	9.413	11.631	11.532
	0.3		9.504	9.402	11.432	11.312
	0.5		9.595	9.481	11.468	11.363
		0.3	9.444	9.417	11.446	11.309
		0.5	9.475	9.489	11.473	11.346

**Table 7:** Percentage augmentation in transmission rate of heat for augmenting values of volumetric fraction.

$\phi_{1},\phi_{2}$	$\begin{array}{l} -Nu\\ TiO_{2}+Ag \end{array}$	$\begin{array}{l} \left(\alpha >0\right)\\ \% \end{array}$	$\begin{array}{l} -Nu\;\\ TiO_{2} \end{array}$	$\begin{array}{l} \left(\alpha >0\right)\\ \% \end{array}$	$\begin{array}{l} -Nu\;\\ TiO_{2}+Ag \end{array}$	$\begin{array}{l} \left(\alpha <0\right)\\ \% \end{array}$	$\begin{array}{l} -Nu\\ TiO_{2} \end{array}$	$\begin{array}{l} \left(\alpha >0\right)\\ \% \end{array}$
0.0	9.232	……	9.232	……	11.142	……	11.142	……
0.01	9.41571	1.995	9.33102	0.907	11.393	2.247	11.302	1.427
0.02	9.52103	3.136	9.42310	2.076	11.543	3.596	11.441	2.678
0.03	9.63142	4.332	9.50211	2.931	11.684	4.864	11.573	3.868
0.04	9.74532	5.566	9.61312	4.134	11.804	5.933	11.679	4.818

## Data Availability

All data used in this manuscript have been presented within the article.
